# A web-based computer-tailored smoking prevention programme for primary school children: intervention design and study protocol

**DOI:** 10.1186/1471-2458-12-277

**Published:** 2012-06-11

**Authors:** Henricus-Paul Cremers, Liesbeth Mercken, Anke Oenema, Hein de Vries

**Affiliations:** 1 Department of Health Promotion, School for Public Health and Primary Care (CAPHRI), Maastricht University, P.O. Box 616, 6200, MD, Maastricht, The Netherlands

## Abstract

**Background:**

Although the number of smokers has declined in the last decade, smoking is still a major health problem among youngsters and adolescents. For this reason, there is a need for effective smoking prevention programmes targeting primary school children. A web-based computer-tailored feedback programme may be an effective intervention to stimulate youngsters not to start smoking, and increase their knowledge about the adverse effects of smoking and their attitudes and self-efficacy regarding non-smoking.

**Methods & design:**

This paper describes the development and evaluation protocol of a web-based out-of-school smoking prevention programme for primary school children (age 10-13 years) entitled ‘Fun without Smokes’. It is a transformation of a postal mailed intervention to a web-based intervention. Besides this transformation the effects of prompts will be examined. This web-based intervention will be evaluated in a 2-year cluster randomised controlled trial (c-RCT) with three study arms. An intervention and intervention + prompt condition will be evaluated for effects on smoking behaviour, compared with a no information control condition. Information about pupils’ smoking status and other factors related to smoking will be obtained using a web-based questionnaire. After completing the questionnaire pupils in both intervention conditions will receive three computer-tailored feedback letters in their personal e-mail box. Attitudes, social influences and self-efficacy expectations will be the content of these personalised feedback letters. Pupils in the intervention + prompt condition will - in addition to the personalised feedback letters - receive e-mail and SMS messages prompting them to revisit the ‘Fun without Smokes’ website. The main outcome measures will be ever smoking and the utilisation of the ‘Fun without Smokes’ website. Measurements will be carried out at baseline, 12 months and 24 months of follow-up.

**Discussion:**

The present study protocol describes the purpose, intervention design and study protocol of ‘Fun without Smokes’. Expectations are that pupils receiving tailored advice will be less likely to smoke after 24 months in contrast to pupils in the control condition. Furthermore, tailored feedback letters and prompting is expected to be more effective than providing tailored feedback letters only.

**Trial registration:**

Dutch Trial Register NTR3116

## Background

Smoking among children and young adolescents remains a public health problem, causing illness or chronic diseases such as cancer and cardiovascular diseases in older ages [1-3]. Recent national prevalence rates showed that 12% of 12 year old children tried to smoke a cigarette. At the age of 13, this percentage has doubled [4]. Smoking among this age group is a well recognised predictor of later regular smoking, since addiction can occur among 60-90% of those who have smoked only a few cigarettes [5,6]. The earlier people start smoking, the higher their chances of becoming a regular smoker and the more difficult it becomes to quit [7].

Many authors state that smoking prevention programmes should aim at preventing or delaying the start of tobacco use [8], implying the need to target children between the ages of 10 and 12 years [9]. Children increasingly start to experiment with smoking in the transition period from primary to secondary school [10]. Prevention programmes should focus on increasing knowledge and positive attitudes towards non-smoking (anti-smoking attitudes), self-efficacy and skills to refuse cigarettes among children who have never smoked and preventing those who are already experimenting from continuing to smoke [11,12].

Several primary prevention programmes have been designed to discourage experimentation with cigarettes and to deter regular use, mostly delivered in school settings [13]. However, results relating to the effectiveness of such in-school programmes have been mixed. Those that found effects usually report very small reductions in smoking behaviour [13-15]. Additionally, in-school programmes have several drawbacks such as limited time and insufficiently trained or unmotivated personnel [16,17]. A need for out-of-school interventions for youngsters has been articulated in several studies [9,18,19]. Such out-of-school approaches may provide an alternative strategy for youth health promotion [20,21]. Ausems and colleagues [9,22,23] compared the effectiveness of an in-school smoking prevention programme with an out-of-school programme among children aged 10-13 years in the ‘Octopus’ study. In the out-of-school intervention pupils were asked to complete a questionnaire in school, after which they received a print-delivered computer-tailored advice targeting attitude, social influence and self-efficacy at home. The out-of-school intervention showed significant short-term preventive effects on smoking initiation and continuation [9]. However, this was a print-delivered programme, while web-based programmes become more popular are more flexible and may appeal more to children and adolescents. Since there is a lack of web-based smoking prevention interventions, the present study will adapt the effective out-of-school intervention of the ‘Octopus’ study and translate it into a web-based out-of-school version entitled ‘Fun without Smokes’.

Web-based computer-tailored interventions have several benefits, such as easy accessibility, filling out the questionnaire at a suitable time, reading the tailored advice whenever the participant chooses and being cost-effective [24]. However, often high drop-out and discontinuation rates are observed for web-based interventions [25,26]. Repetition of messages (multiple tailoring) seems important in enhancing effectiveness of computer-tailored interventions in general and for smoking prevention specifically [9,22,27,28]. Therefore, it is important to find ways to stimulate participation and promote multiple exposure to educational content.

One means of promoting repeated exposure and preventing drop-out of a programme is to prompt or remind people of their participation or encourage them to revisit a website to gather new information concerning a topic of interest [29]. Prompts can be delivered in several ways, such as through mail, e-mail or short text messages (SMS). The use of prompts to support adherence to health interventions has been shown to be effective in increasing participation rates [30]. Therefore, e-mail and SMS prompts are the chosen methods of promoting repeated use of the intervention website and evaluated for their effects on the use of the programme and the smoking related outcomes.

This article describes the intervention development and the evaluation protocol of ‘Fun without Smokes’.

## Methods & design

### The intervention

#### Intervention objectives

The objective of the intervention is to prevent smoking among primary school children aged between 10 and 13 years. To achieve this, children will be provided with personalised feedback including information about non-smoking, positive attitudes towards non-smoking, negative attitudes towards smoking and skills and plans to refuse cigarettes. This feedback will prepare children to refuse cigarettes when they make the transition to secondary school and prevent them from becoming a (regular) smoker. E-mail and SMS messages will be used as prompts to promote visits to the intervention website, where children are exposed to non-smoking related information and can receive repeated tailored feedback letters.

#### Intervention framework and procedures

‘Fun without Smokes’ is an out-of-school smoking prevention programme, in which children receive computer-tailored feedback letters and can visit a website that provides additional information about non-smoking. The website contains a section in which children can complete an assessment questionnaire to receive computer-tailored feedback. They can also access additional information about non-smoking, watch short movies about (the consequences of) smoking, play a game or ask questions concerning (non-)smoking. These sections will be regularly updated to stimulate the pupils to revisit the ‘Fun without Smokes’ website.

Children first visit the website to complete the assessment questionnaire. This first assessment will take place in the class-room. Based on this questionnaire three personalised feedback letters are generated that are posted on the website in a password protected area and that are also sent as pdf attachments to the participants e-mail address. Thus, the children can read their personalised feedback letters either as pdf attachment and/or at the ‘Fun without Smokes’ website. The feedback letters are sent on three consecutive days. The first letter appears one hour after completion of the assessment questionnaire in the e-mail or at the website.

**Figure 1:**
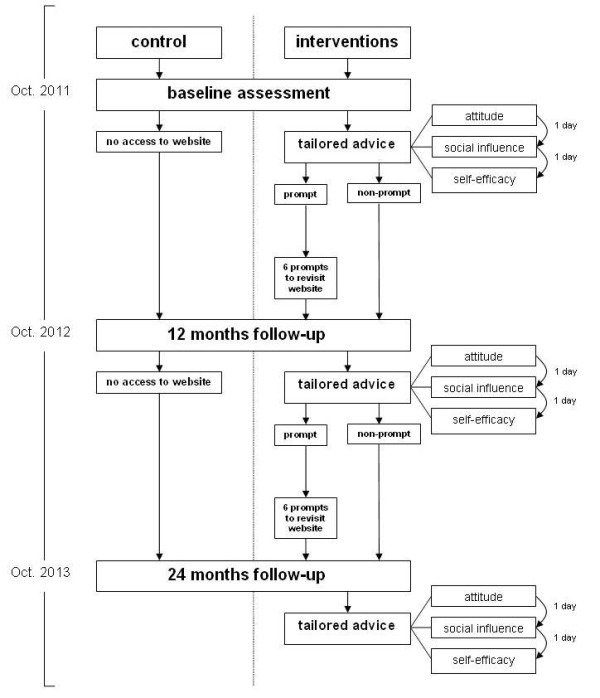
Design of ‘Fun without Smokes’.

In order to allow the most flexible adaptations to changes in children’s lives, pupils in the intervention conditions also have the opportunity to complete additional questionnaires between the measurement periods. To do so, they can return the ‘Fun without Smokes’ website and choose the option to complete a questionnaire and receive additional feedback letters. Participants are able to choose which set of questions they want to fill out (i.e., attitude, social influences or self-efficacy). If one of these smaller questionnaires is completed a feedback letter is generated, that is sent as pdf attachment to the respondents e-mail address and is posted on the website. To prompt children to revisit the website, participants in the intervention + prompt condition will receive SMS and e-mail messages. Figure 1 shows the design of the ‘Fun without Smokes’ study with the measurement periods during the intervention trial.

All participants will be invited to complete the full assessment questionnaire again 12 and 24 months after the baseline questionnaire is completed, to receive repeated feedback letters about changes in smoking status or key determinants (e.g. attitude, social influences and self-efficacy expectations). However, the tailored advice will take into account the answers of the pupils of the previous measurements.

To increase the probability that the intervention will be attractive, 87 pupils from grade 7 and 8 (10-12 year olds) were involved in the development process. Group interviews were performed to select the name, colour scheme and design for the intervention and website. After developing several lay-outs, websites and logos these were presented to children and they were asked to choose their favourite name. ‘Fun without Smokes’ came out as the most popular. Additionally, short animations were developed to make completion of the questionnaire more attractive. In those animations the consequences of smoking were depicted by animals. Children appreciated these animations.

#### Theoretical background

**Figure 2:**
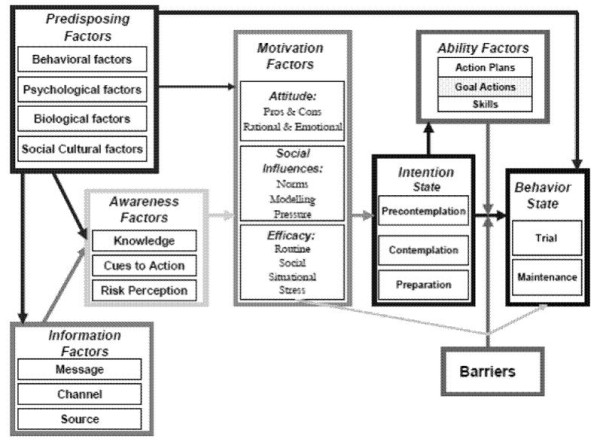
I-Change model.

The tailored feedback messages provided in the ‘Fun without Smokes’ programme are developed based on the integrated model for exploring motivational and behavioural change (I-Change Model), a model composed of an integration of various social cognitive theories and models [31-33]. According to the I-Change Model (Figure 2), behaviour is determined by people's motivation or intention to perform a certain behaviour, but barriers can decrease the chance that intentions will translate into action. An individual’s abilities, such as being able to plan specific actions to reach the target behaviour (i.e., action plans), can increase the chance that intentions will translate into action. Motivational factors, such as attitude, social influences and self-efficacy expectations determine a person’s intention to change. These motivational factors are influenced by awareness factors, for example, knowledge or cues-to-action, information factors, such as message quality, and predisposing factors, namely behavioural, psychological, biological, social and cultural factors [32]. The I-Change Model is used in this study to develop the questionnaire and computer-tailored advice.

#### Content of the tailored feedback letters

Pupils in the intervention groups receive three computer-tailored feedback letters after completing the baseline assessment questionnaire. The main determinants from the I-Change Model (attitude, social influences, self-efficacy expectations, intention and behaviour) are addressed in the tailored feedback letters by using theoretical methods that promote changes in these determinants [34,35].

The first tailored feedback letter is focussed on knowledge and attitudes. To improve knowledge, a knowledge quiz about smoking was included in the questionnaire and the score to this quiz was fed back in the letters. Pupils with a high score on the knowledge quiz receive a compliment for their good knowledge. The correct answer is provided and clarified (elaboration) for questions that are answered incorrectly. Additionally, advice about pupils’ attitudes towards (non-)smoking was provided in this first tailored feedback letter. Attitude was addressed by confirming non-smoking attitude beliefs and providing arguments to counter pro-smoking beliefs.

The second tailored feedback letter addresses subjective norm and social influence. Feedback was given on perceived norms and actual smoking behaviour by significant others such as mother, father or siblings (information about others’ approval). These letters provided information on the number of people that smoke in the Netherlands, to indicate smoking is not the norm. Furthermore, strategies were provided for how to deal with unsupportive social circumstances (e.g. when parents and siblings smoke) and how to mobilize support for non-smoking (stimulate communication to mobilise social support).

Self-efficacy is the focus of the third and final tailored feedback letter. When pupils were confident that they would be able to stick to their no smoking intentions and to refuse a cigarette, even in difficult situations (e.g. when friends offer you a cigarette) the feedback was focussed on strengthening their confidence. When pupils lower self-efficacy expectations the feedback provided strategies, tips and tricks for how to deal with difficult situations (verbal persuasion/exhortation). In addition, information was given about the formation of action plans. Pupils were motivated to form a plan for how to refuse a cigarette in a situation that they thought would be difficult (cue identification to form action plans for difficult situations). An overview of the determinants, theoretical methods and examples used in the ‘Fun without Smokes’ intervention is shown in Table 1.

**Table 1:** Overview of methods in computer-tailored messages

**Determinant**	**Method(s)**	**Examples in ‘Fun without Smokes’ intervention**
**Knowledge**	· Elaboration	How much do you know about smoking? If we have to give you a score you get a **7**. That is a really good start! You can read here what you didn’t know yet:
		→smoking is harmful, also when you smoke once in a while
		→smokers have to cough more often to get rid of the toxic substances in their body
**Attitude**	· Arguments	You think that you won’t get nauseous if you smoke. But that is not right. When you smoke, there will be less oxygen in your blood. This can make you very nauseous.
**Social influence (norms/modeling)**	· Information about others’ approval	Both your mum and dad think you should not smoke. Maybe you don’t want to listen to your parents. But they are right! They will certainly support you not to smoke. Maybe you can arrange a nice present if you decide not to smoke.
	· Stimulate communication’ to mobilize social support	
**Self-efficacy**	· Verbal persuasion/exhortation	If one of your friends offers you a cigarette, it is difficult for you not to smoke. Towards a friend you can say you don’t want to smoke. Keep they pressing you to smoke? Than you start talking about something else. Such as a new movie, a nice computer game or a new television show you recently saw.
	· Planning coping responses	
**Formation of action plans**	· Cue identification to form action plans for difficult situations	It is very smart of you that you don’t want to smoke. But what if something unexpected happens? For instance, you are at a party and it is very sociable. Some of your friends light up a cigarette. And they also offer you one. It is difficult to refuse the cigarette. What would you do?
		Our tip is to think in advance what you would do in such an unexpected situation. Now imagine for yourself a plan not to smoke. A few examples are:
		-I make a plan with myself that I will never smoke
		-I ignore people if they offer me a cigarette.
		-I will talk about something else.
		-I just say ‘no’ if friends offer me a cigarette.
		-I keep saying ‘no’ also when friends being obtrusive.
**Intention to change**	· Re-evaluation of intention to change behavior	You don’t know if you want to start smoking. However, you want to try a cigarette sometime in the future. Maybe you think this is exciting, but do you think this is wise? After reading this letter you probably think different about smoking.

#### Pre-test of the intervention

Before the start of the intervention a pre-final version of the programme was pre-tested among a small group of children from the target group. Two pre-tests were performed to determine the usability of the website, accessibility to the questionnaire and the complete procedure at primary schools. Techsmith Morae software [36] was used to test the usage of the ‘Fun without Smokes’ website and the web-based questionnaire among 5 children (aged 10-11 years) and 2 teachers. To examine possible bottlenecks at the school level for the ‘Fun without Smokes’ programme, a pilot test was performed in two primary schools (26 children). The complete procedure within the school was tested: logging in at the website, searching the questionnaire, completing the questionnaire and reading the personalised advice. Afterwards, qualitative and quantitative interviews were carried out to obtain detailed information about the duration of completing the questionnaire, procedure in the classrooms and the readability of the personalised advice. Based on the results minor adjustments were made to improve the readability of the personalised advice and to simplify the log out procedure of the website.

### Evaluation design

#### Objectives and design

Both versions of the ‘Fun without Smokes’ programme (intervention and intervention + prompt condition) will be evaluated in a cluster randomised controlled trial with a no information control condition. The aim is to test the effectiveness of the programme on prevention of smoking initiation and on cognitions in favour of non-smoking at 12 and 24 months post baseline, as well as the additional effect of prompts on the usage of the website and on the outcome measures. Schools were the units of randomisation.

Assessment of smoking behaviour and smoking related cognitions will be at baseline and 12 and 24 months later, by means of web-based questionnaires.

Ever smoking is chosen as the primary outcome measure, because prevalence rates of monthly smoking are generally low at the age of 13 (8% at this age in 2010 [4]). Additionally, the level of utilisation of the website will be a primary outcome measure. This outcome will be operationalized as the number of visits to the ‘Fun without Smokes’ website and is expected to be significantly higher in the intervention + prompt condition compared with the intervention condition. Secondary outcome measures are attitudes, social influences, self-efficacy, intention to smoke and monthly smoking.

The hypotheses for this study are:

1. Providing children with computer-tailored feedback about non-smoking will prevent smoking initiation;

2. Children receiving computer-tailored feedback about non-smoking will have stronger cognitions in favour of non-smoking;

3. Computer-tailored feedback about non-smoking and prompting children through e-mail and SMS to revisit the website will result in a stronger effect on the prevention of smoking initiation (and on cognitions in favour of non-smoking) than tailored feedback only;

4. Children receiving computer-tailored feedback, SMS and e-mails will visit the website more often than children receiving computer-tailored feedback only.

#### Sample size calculation

Based on a sample size calculation 81 school and 3,240 pupils need to be included in the study. This calculation is based on the assumptions that 15% of the intervention condition and 8% of the intervention + prompt condition have ever smoked 24 months after baseline, whereas among the control condition the national prevalence rate of ever smoking is expected to be 24% at the age of 13. We used the OD (Optimal Design) method of Raudenbush [37] with a two-sided significance level of 0.05, a target power of 0.80 and an ICC of 0.04 (based on the study of Ausems et al. [38]) to calculate the sample size.

To calculate the sample size for the utilisation of the website 300 children per condition will be needed (600 children in total). It is assumed that pupils in the intervention + prompt condition will revisit the ‘Fun without Smokes’ website more often, with a mean usage of 5.5 (SD = 0.8). In contrast, children in the intervention only condition will visit the website at least three times (baseline, 12 months and 24 months) and a mean usage of 3.5 is expected.

#### Randomisation

Schools are the unit of randomisation and will be randomly assigned to one of the three study conditions in a computer determined sequence using a clustered randomisation scheme.

#### Utilisation of the website

Use of the ‘Fun without Smokes’ website will be monitored by using web-logs of the intervention and the intervention + prompt condition. Every participant has a personal code (username). Based on this username web-logs can identify how often and when (date) a participant visits the website as well as which website components (e.g. reading tailored feedback letters, reading additional information, playing games, completing the questionnaire) he or she clicks.

#### Recruitment procedure

**Figure 3:**
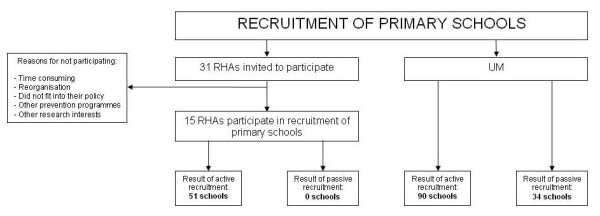
Recruitment of primary schools.

‘Fun without Smokes’ will be implemented in primary schools as a new programme offered via Regional Health Authorities (RHAs) in the Netherlands. A total of 31 RHAs in the Netherlands were invited through e-mail and telephone to participate in the recruitment of primary schools within their own region. Nine RHAs decided to participate in the active recruitment (information via telephone or school visits) and six were able to recruit primary schools in a passive manner (information via e-mail or newsletters). Maastricht University (UM) performed the same method in the regions of non-participating RHAs. Schools from all regions in the Netherlands will be included to reach a diverse sample of pupils. The intervention will be presented as an out-of-school programme, since the main part of the intervention takes place outside the school system. Over 3,500 Dutch primary schools were contacted for participation and 175 schools were willing to take part in the study (effectiveness rate = 5.0%). This procedure is illustrated in detail in Figure 3.

The criteria for inclusion are that primary schools want their final two grades (pupils aged 10-12 years) to participate in the study and the children have computers with Internet access at school and at home. Special education schools and schools using the prevention programme ‘I don’t smoke (either)’ [‘ik (r)ook niet’] were excluded. Schools that were interested in participation received an invitation letter and information brochure via the RHAs or UM. Furthermore, the programme was explained via telephone to the school principal. After two weeks, they were re-contacted by a member of the research team to discuss participation in the effectiveness study. When schools were interested in participation, agreement with inclusion criteria was checked.

Parents and children were informed about the procedure and goals of the study by means of a letter that children could take home for their parents. All students in grade seven of a participating school were included in the study unless they or their parents refused to be involved. By signing the informed consent letter and returning this to the research team, parents and children were able to deny participation before the start of the study. Additionally, they were able to withdraw participation at any moment by e-mail, telephone or in person during the entire study period.

#### Procedure

The baseline questionnaire (which is also the screening instrument for the tailored feedback messages) was completed during school hours under the supervision of a teacher, who also distributed to all participating children their personal log-in details in sealed envelopes. Non-participating children were instructed by the teacher to do something else. The same procedure will be employed for the one year follow-up assessment. The two year follow-up questionnaire will have to be completed at home, since by that time the children will have left primary school. For the two year follow-up the participants will be contacted via mail and e-mail inviting them to complete the web-based questionnaire.

After completion of the baseline and 12 months questionnaire, the children in the intervention groups will receive short summaries of the tailored feedback on their computer screen. Extended tailored advice will be sent one hour after completion of the web-based questionnaire as pdf attachment to the respondents e-mail address and will be posted on the website. In the next 12 months, children in the intervention groups can visit the website, to obtain new information or receive new advice as often as they want.

Six times a year participants in the intervention + prompt condition will be pro-actively prompted through e-mail and SMS to revisit the ‘Fun without Smokes’ website. The first three prompts will be sent 1, 2 and 3 months after the baseline measurement. The last three prompts will be sent 5, 7 and 9 months after baseline. In accordance with the prompts, some of the content of the website will be refreshed to address a new topic relevant for smoking prevention. This procedure will be repeated in the second year of the evaluation study. Participants in the intervention condition will not receive these messages, but will be able to revisit the website whenever they want during the 2-year follow-up period to view the same information as shown to participants in the intervention + prompt condition. The participants in the control condition will not have access to the ‘Fun without Smokes’ website.

#### Questionnaire

The web-based questionnaire of the ‘Fun without Smokes’ program is an adapted version of the ‘Octopus’ questionnaire that assesses the predictors of smoking [9,22,23,38]. *Demographics* will be assessed in terms of: age, gender, name and location of the school, religion, ethnicity, family composition, pocket money, school performance, mobile phone number, e-mail address and socioeconomic status (SES). SES will be measured using the Family Affluence Scale (FAS), a scale to measure family wealth based on social and economic markers [39,40]. The FAS includes four questions concerning car ownership, number of holiday trips a year, number of computers at home and whether the respondent has his or her own bedroom. Answers will be combined in an index.

*Knowledge* of smoking will be assessed with six questions such as ‘When you smoke you have to cough more often’. The answer category includes ‘yes’, ‘no’ and ‘I don’t know’.

*Attitude* will be assessed using nine advantages (pros) and ten disadvantages (cons) of smoking. Participants have to complete the question ‘If I smoke…’ with answers such as ‘I will feel very mature - I will feel not mature’ (pro) or ‘I will become very ill - I will not become ill’ (con) on a four point scale. This procedure has also been supported in previous studies and indicated reliable scales [41].

*Social influence* will be measured using two different concepts: social norms and modelling. *Social norms* assess beliefs about smoking among parents, family and friends. Participants have to complete questions such as ‘My mother thinks that I…’ with a five point answering scale ranging from ‘definitely should not smoke’ to ‘definitely should smoke’. *Modelling* includes the smoking behaviour of parents, family and friends. In total, five questions will be included such as ‘Does your mother smoke?’ with a five point answer scale ranging from ‘often’ to ‘never’. The number of smokers in their environment will be assessed with three questions, for example, ‘How many of your friends smoke?’ a five point scale is used as answering option (almost all - almost none).

*Self-efficacy* expectations will be assessed using ten items, in order to determine how easy or difficult it would be not to smoke in various situations. The questions are answered on a five point scale ranging from ‘very easy’ to ‘very difficult’ not to smoke [42]. ‘When others smoke it is…..for me not to smoke’ or ‘When my parents offer me a cigarette it is…for me not to smoke’ are examples of self-efficacy questions.

*Intention* will be measured differently for smokers and non-smokers. One question will assess smokers’ intention to stop smoking whereas non-smokers will be asked if they want to start (experimenting with) smoking (two questions). Intention will be measured on a five point scale with answering options ranging from ‘definitely yes’ to ‘definitely not’.

*Action planning* will also be differentiated for smokers and non-smokers. Smokers will be able to assess action plans on how to stop smoking, whereas non-smokers will be able to assess ways to refuse cigarettes. For both groups, action plans will be measured by five items, such as ‘I throw my cigarettes and lighter away’ for smokers or ‘I refuse that cigarette’ for non-smokers. These questions can be completed on a five point scale ranging from ‘totally agree’ to ‘totally disagree’.

*Smoking Behaviour* will be assessed by self-reports with the question: ‘Which statement describes your behaviour best?’ An internationally accepted algorithm [9,11,43,44] will be used as answering scale, ranking the following items: ‘I have never smoked, not even a puff’, ‘I have tried smoking once, but I do not smoke anymore’, ‘I have quit smoking, I have always smoked less than once a week’, ‘I have quit smoking after having smoked at least once a week’, ‘I try smoking once in a while’, ‘I smoke less than once a month’, ‘I do not smoke weekly, but at least once a month’, ‘I do not smoke daily, but at least once a week’ and ‘I smoke at least once a day’. Children will be categorised as non-smokers (never smoked a puff; have tried smoking, but not anymore; stopped smoking, after smoking less than once a week; stopped smoking, after smoking at least once a week) or as smokers (try smoking sometimes; smoke less than once a month; smoke at least once a month; smoke at least once a week; smoke every day). Smoking during the last 24 hours (‘Have you smoked during the last 24 hours?’), the past 7 days (‘Have you smoked during the past 7 days?’) and during the past month (‘Have you smoked during the past month?’) will be assessed as well. These questions will be assessed with a ‘yes/no’ answering scale. Additionally, a qualitative assessment will be made of the number of cigarettes smoked during the past week and weekend (‘How many cigarettes have you smoked during the last week/weekend?’).

#### Process evaluation

Pupils in both the intervention conditions will receive a process evaluation questionnaire to assess the tailored feedback letters. An invitation to fill out this (brief) web-based questionnaire will be sent via e-mail one day after they have received their third feedback letter. Children will be asked to indicate how many letters they have read and give an overall rating (1 = low to 10 = high) of the individual feedback letters. The process evaluation questionnaire will, furthermore, assess other aspects of appreciation, such as how attractive, pleasurable and comprehensible they thought the provided feedback was. These questions can be answered on four point scales. Examples of answering options are level of attractiveness (very nice - not nice), comprehensibility (very clear - not clear) and level of personalisation (very personal - not personal). After 12 and 24 months pupils in both the intervention conditions will be asked how many prompts they received (SMS and e-mail). Their opinions about these prompts will also be assessed. The amounts of visits will be qualitatively assessed by asking the pupils if they visited the ‘Fun without Smokes’ website and if they noticed the updates on the website. In addition, 12 and 24 months after baseline telephonic in-depth interviews will be held to assess qualitatively the evaluation of the programme among pupils.

#### Statistical analyses

Multilevel logistic regression analyses will be used (MLwiN) to examine differences between the three study groups on smoking status and cognitions in favour of (non-)smoking. Mediation and moderation analyses will be performed to identify cognitive factors as mediators of the intervention effect and to explore differential effects in sub-groups, for example, based on demographic characteristics. Data of process evaluation interviews held after 12 and 24 months among pupils will be analysed using Nvivo.

#### Ethical approval

This study is approved by the Medical Ethics Committee of the Atrium-Orbis-Zuyd Hospital (NL32093.096.11/MEC 11-T-25) and registered in the Dutch Trial Register (NTR3116).

## Discussion

In this paper the design of the intervention and evaluation protocol of the ‘Fun without Smokes’ programme has been presented.

The present study aims to develop a computer-tailored smoking prevention programme for primary school children, in which they will be provided with the necessary attitudes, knowledge, skills and plans to refuse cigarettes when they make the transition from primary to secondary school. The programme is an adaptation and translation of an existing computer-tailored intervention that was previously print-delivered. The content of the computer-tailored feedback was similar to the original print-delivered version, but the messages are much shorter to comply with the requirements that messages through the Internet should not be too long. The time interval of sending the feedback letters was made shorter (one day as opposed to two weeks in the print-delivered version), since this was preferred by the pupils. Furthermore, the web-based delivery allowed for the creation of a more comprehensive website where children could find more information about (non-)smoking, games and additional movies concerning (non-)smoking. This website was used to facilitate multiple exposure to non-smoking information and to be able to provide multiple feedback.

The structural approach - involvement of pupils, using a medium preferred by pupils and pre-testing of the programme - is a good basis for an effective intervention and increases the likelihood that the web-based approach is attractive to pupils. Two versions of the intervention will be tested in a three-group cluster randomised controlled trial. It is hypothesised that pupils in the intervention conditions will be less likely to start (experimenting with) smoking compared with the control condition, and that the smoking rates will be lower in the intervention + prompt condition than the intervention condition.

### Strengths and limitations

Previous in-school smoking prevention programmes required much investment in time from the participating (primary) schools [45], leading to frustration and higher drop-out rates of participants or schools. The ‘Fun without Smokes’ programme is a web-based intervention, which requires only a very limited input and time from teachers, and thus meets the needs of schools to lower workloads involved in implementing smoking prevention programmes. A second strength is the design of the effectiveness study, since it assesses effects not only immediately, but also after 12 and 24 months. This allows testing both the short- and long-term effects of the ‘Fun without Smokes’ programme.

A possible limitation of the study is that smoking behaviour and other factors related to smoking are based on self-reports of youngsters, which might lead to measurement errors (i.e. social desirable answers). To avoid social desirable answers, we will guarantee full confidentiality to our participants. The validity of adolescent self-reported smoking has been shown to be high in accordance with biological indicators when measurement assures confidentiality [46]. Approximately 60% is expected to drop-out in the final measurement, since pupils are not connected to their primary school anymore. This drop-out percentage is taken into account in the sample size calculation. The large sample size will guarantee enough power for the evaluation study.

If the programme is proven to be effective, it will be a valuable tool for smoking prevention that can readily be implemented in primary schools in the Netherlands. This study will, furthermore, provide valuable information about the usability and additional effects of SMS and e-mail reminders in combination with a computer-tailored feedback programme.

## Misc

Henricus-Paul Cremers, Liesbeth Mercken, Anke Oenema and Hein de Vries contributed equally to this work.

## Competing interests

HdV is scientific director of Vision2-Health, a company that licenses evidence-based innovative computer-tailored health communication tools.

## Authors’ contributions

HdV designed and wrote the original proposal. HPC, LM, AO and HdV developed the smoking prevention programme and execute the studies. HPC significantly contributed to writing this paper, while LM, AO and HdV were involved in revising the manuscript critically. All authors read and approved the final version of the manuscript.

## Pre-publication history

The pre-publication history for this paper can be accessed here:

http://www.biomedcentral.com/1471-2458/12/277/prepub
